# The Cytokinin Complex Associated With *Rhodococcus fascians:* Which Compounds Are Critical for Virulence?

**DOI:** 10.3389/fpls.2019.00674

**Published:** 2019-05-22

**Authors:** Paula E. Jameson, Pragathi Dhandapani, Jiancheng Song, Marek Zatloukal, Miroslav Strnad, Mitja N. P. Remus-Emsermann, Rudolf O. Schlechter, Ondrej Novák

**Affiliations:** ^1^School of Biological Sciences, University of Canterbury, Christchurch, New Zealand; ^2^School of Life Sciences, Yantai University, Yantai, China; ^3^Laboratory of Growth Regulators, The Czech Academy of Sciences, Faculty of Science, Institute of Experimental Botany & Palacký University, Olomouc, Czech Republic, China

**Keywords:** dimethyl transferase, fasciation, isopentenyl transferase, methylated cytokinin, *Williamsia*

## Abstract

Virulent strains of *Rhodococcus fascians* cause a range of disease symptoms, many of which can be mimicked by application of cytokinin. Both virulent and avirulent strains produce a complex of cytokinins, most of which can be derived from tRNA degradation. To test the three current hypotheses regarding the involvement of cytokinins as virulence determinants, we used PCR to detect specific genes, previously associated with a linear virulence plasmid, including two methyl transferase genes (*mt1* and *mt2*) and *fas4* (dimethyl transferase), of multiple strains of *R. fascians*. We inoculated *Pisum sativum* (pea) seeds with virulent and avirulent strains of *R. fascians*, monitored the plants over time and compared these to mock-inoculated controls. We used RT-qPCR to monitor the expression of *mt1, mt2*, and *fas4* in inoculated tissues and LC-MS/MS to obtain a comprehensive picture of the cytokinin complement of inoculated cotyledons, roots and shoots over time. The presence and expression of *mt1* and *mt2* was associated with those strains of *R. fascians* classed as virulent, and not those classed as avirulent. Expression of *mt1, mt2*, and *fas4* peaked at 9 days post-inoculation (dpi) in cotyledons and at 15 dpi in shoots and roots developed from seeds inoculated with virulent strain 602. Pea plants inoculated with virulent and avirulent strains of *R. fascians* both contained cytokinins likely to have been derived from tRNA turnover including the 2-methylthio cytokinins and *cis*-zeatin-derivatives. Along with the isopentenyladenine-type cytokinins, the levels of these compounds did not correlate with virulence. Only the novel 1- and 2-methylated isopentenyladenine cytokinins were uniquely associated with infection by the virulent strains and are, therefore, the likely causative factors of the disease symptoms.

## Introduction

*Rhodococcus fascians* (Tilford, 1936; Goodfellow, 1984) is a soil-borne, Gram positive Actinobacterium, some strains of which are reported to be growth-promoting epiphytes (Francis et al., 2016; Savory et al., 2017), whereas other strains have the ability to exist not only as epiphytes but also can invade a wide variety of host plants and exist as endophytes causing fasciation symptoms (Lacey, 1939; Miller et al., 1980; Savory et al., 2017). Virulent bacteria often feature different life styles and may be asymptomatic leaf surface colonizers, epiphytes, before they invade the apoplast and feature an endophytic lifestyle. This movement toward the endophytic compartment may be coordinated by quorum sensing decisions (Dulla and Lindow, 2008) which may also apply to *R. fascians* (Stes et al., 2013). The majority of the pathogenic endophytic strains of *R. fascians* harbor a linear plasmid which is strictly associated with virulence (Crespi et al., 1992; Stange et al., 1996; Savory et al., 2017). Fasciation symptoms of sweet peas caused by virulent strains are characterized by a release from apical dominance, the outgrowth of swollen fleshy lateral stems (Lacey, 1936; Tilford, 1936), reduced shoot and leaf growth (Lacey, 1936) and reduced root growth (Oduro and Munnecke, 1975). Cotyledons in garden peas inoculated by virulent strains do not enter senescence (Oduro and Munnecke, 1975; Eason et al., 1995; Dhandapani et al., 2017). Leafy galls induced following inoculation of Arabidopsis by *R. fascians* consist of multiple, partially expanded shoots that have arisen from existing and newly induced meristems (Vereecke et al., 2000; de O Manes et al., 2001; Depuydt et al., 2008). However, the symptoms displayed can depend on the method of inoculation (Goethals et al., 2001; Dolzblasz et al., 2018).

The cytokinins had originally been proposed to be the causative factors of the growth abnormalities, as the reduced apical dominance and reduced shoot growth can both be mimicked by cytokinin application (Thimann and Sachs, 1966; Armstrong et al., 1976) and by overexpression of *ipt* (McKenzie et al., 1998). Cytokinins are produced in plants and by several plant-associated bacteria *de novo* via isopentenyl transferase (IPT) linking an ATP/ADP (in plants) or AMP (in bacteria) to an isopentenyl side chain. The first formed isoprenoid cytokinins are considered to be inactive nucleotides, but activated when the ribotide moiety is removed by the enzyme Lonely Guy (LOG) (Kuroha et al., 2009). The isopentenyl side chain may remain non-hydroxylated providing the isopentenyladenine (iP)-type cytokinins, or become hydroxylated leading to the zeatin (*t*Z)-type cytokinins. The double bond in the side chain may be hydrogenated to form the dihydrozeatin (DHZ) derivatives. The adenine moiety may be ribosylated or glucosylated, and the side chain *O*-glucosylated (Sakakibara, 2006). The free bases (iP, Z, DHZ) are considered the biologically active forms (Lomin et al., 2015); the ribosides the predominant mobile forms (Zürcher and Müller, 2016); and the *O*-glucosides are storage forms (Jameson, 1994; Kieber and Schaller, 2018).

Cytokinins are also found associated with specific tRNA molecules of bacteria and all eukaryotes (Perrson et al., 1994; Gajdošová et al., 2011), produced via tRNA-IPTs (Miyawaki et al., 2006). Six tRNA-associated forms have been identified in plants including *cis*- and *trans*-zeatin riboside (*c*ZR and *t*ZR), isopentenyladenosine (iPR), 2-methylthio-iPR (2MeS-iPR), and 2-methylthio*-c*ZR and -*t*ZR (2MeS-*c*ZR and 2MeS-*t*ZR) (Taller, 1994). The *cis*-Z forms are typically the most abundant in plant tRNAs and, when released, potentially have biological roles within the plant (Gajdošová et al., 2011; Schäfer et al., 2015). Twelve different cytokinins (*c*Z, *c*ZR, *t*Z, *t*ZR, iP and iPR, and their 2MeS-derivatives) have been unequivocally identified in culture filtrates of *R. fascians* (Armstrong et al., 1976; Murai et al., 1980; Pertry et al., 2009, 2010; Tarkowski et al., 2010). Hydrolysates of *R. fascians* tRNA released iPR, *c*ZR, 2MeS-*c*ZR, 2MeS-iPR, and 2MeS-iP (Murai et al., 1980).

Confirmation that cytokinins could be synthesized *de novo* by *R. fascians* and were intimately involved in symptom development was shown by the presence of genes on the virulence plasmid associated with cytokinin biosynthesis, variously referred to as *fasD/fas4/ipt*, cytokinin activation (*fasF/log*) and destruction (*fasE/ckx*) (Crespi et al., 1992; Pertry et al., 2010; Francis et al., 2012). Cytokinin oxidase (CKX) removes the side chain and irreversibly inactivates the molecule. Between the various isoforms of CKX (both plant and *fasE*), the cytokinins previously detected in *R. fascians* culture filtrates can be degraded but with different efficiencies: the greatest activity occurs against iP-types whereas 2MeS-*c*Z is nearly resistant to CKX degradation (Pertry et al., 2009, 2010).

Based on the classical Skoog and Miller (1957) model of organogenesis, as well as on the manipulations of the cytokinin and auxin biosynthesis genes of the T-DNA of *Agrobacterium tumefaciens* (Morris, 1987), the shooty galls caused by virulent strains of *R. fascians* would be expected to have higher levels of cytokinin relative to auxin, and certainly substantially greater levels of cytokinin than tissue inoculated with avirulent strains, but this has not routinely been shown to be the case (Eason et al., 1996; de O Manes et al., 2001; Gális et al., 2005; Dhandapani et al., 2017, 2018). It is also important to note that the iP-type cytokinins are secreted into culture medium by both virulent and avirulent strains of *R. fascians* at similar levels to those found in the culture filtrates of other gall-inducing bacteria, while the levels of hydroxylated cytokinins are at least 1,000 times lower, even when *R. fascians* is induced [see (Jameson, 2000)]. Under “inducing” conditions *in vitro*, Pertry et al. (2010) showed increased secretion of five cytokinins by the pathogenic strain D188, but with the greatest increase only being from ca. 2–4.5 nM for 2MeS-*c*Z relative to the plasmid-less strain, whereas induction by acetosyringone of *A. tumefaciens* C58 led to an increase in hydroxylated cytokinins from 0.05 to 35 μM *in vitro* (Powell et al., 1988). However, the presence of the bacteria and the concomitant response of the plant are important factors that need to be taken into account.

To address this issue, and based on levels of cytokinin in Arabidopsis inoculated with virulent strain D188 in comparison to mock-inoculated controls, Pertry et al. (2009) put forward the novel “Trick-with-the-Cytokinin-Mix” hypothesis, suggesting that 2MeS-*c*Z and *c*Z accumulated in Arabidopsis tissues infected with virulent strain D188, because they were poor substrates for CKX, leading to a continuous supply of cytokinin maintaining tissue proliferation. However, neither *c*Z nor 2MeS-*c*Z differentially accumulated in tobacco infected with either virulent strain D188 or 6162 whereas iP did (Pertry et al., 2009; Radhika et al., 2015). It was therefore speculated that, depending on the homeostatic capacities of the host, a subset of the cytokinin mix would be resistant and provoke symptoms (Pertry et al., 2009).

In contrast to the above hypothesis, Creason et al. (2014) suggested that only the iP-type cytokinins are synthesized in a *fas*-dependent manner and necessary for inducing symptoms. Isopentenyladenine has been correlated positively with virulence symptoms in tobacco (Pertry et al., 2009; Radhika et al., 2015) but these values were presented only against mock-inoculated controls and not compared with inoculation with an avirulent strain. When the full comparison is done, while there was a positive correlation between iP-types in pea cotyledons and virulence (Dhandapani et al., 2017), this correlation did not hold as strongly for leaves and roots (Dhandapani et al., 2018).

Based on the finding of Pertry et al. (2009) that knockout mutants of two methyl transferase genes, *mt1* and *mt2*, located upstream of the fasciation (*fas)* operon were non-pathogenic, Vereecke's group hypothesized that methylated cytokinins might be involved in disease induction (Stes et al., 2011; Francis et al., 2012). Subsequently, Radhika et al. (2015) identified two novel methylated cytokinins produced by strain 6162 (ATCC 35014), and showed that the two methyl transferases synthesized methylated side chains, the dimethyl form of which was utilized by Fas4 to produce 2-MeiP. This biologically active cytokinin inhibits root growth, is recognized by cytokinin receptors and was shown to be as resistant to degradation by AtCKX2 as *c*Z *in vitro*. While the levels of 2-MeiP were still in the pmol/g fresh weight range, and at similar levels to iP, *c*Z, and *t*Z in infected tobacco tissue, the methylated cytokinins were not detected in uninfected tissue (Radhika et al., 2015). Radhika et al. (2015) showed that *E. coli* expressing just *mt1, mt2*, and *fas4* were sufficient to produce 2-MeiP. They concluded that MT1 and MT2 were not involved in the synthesis of the methylthio-derivatives of cytokinins as suggested by Pertry (2009), and proposed that Fas4 is a dimethyl transferase, rather than an isopentenyl transferase, and produces the long sought after cytokinin signal.

Garden pea (*Pisum sativum*) has been used as a model system for *R. fascians* infection for many years (Oduro and Munnecke, 1975). We have shown in cotyledons of *R. fascians* inoculated pea that *PsIPT* is expressed and that cytokinin signaling is activated in a very early response to the presence of both virulent and avirulent strains of *R. fascians*—but signaling is activated more strongly to the virulent strain (Dhandapani et al., 2017). Moreover, cytokinin signaling, as shown by upregulation of cytokinin response regulator (*PsRR)* expression, is then only maintained in the cotyledons infected by the virulent strain. However, we showed that neither the standard zeatin-type nor the iP-type cytokinins correlated well with symptom development following comparison of cytokinins in pea cotyledons, shoots and roots in mock-inoculated controls and after inoculation with a virulent and an avirulent strain of *R. fascians* (Dhandapani et al., 2017, 2018). To further investigate the three hypotheses outlined above, in this paper we present a comprehensive analysis of the *cis*-forms and the 2-methylthio cytokinins (2MeS-CKs), along with expression of *mt1* and *mt2* and the measurement of 1- and 2-MeiP in cotyledons, shoots and roots of pea inoculated with both the virulent and avirulent Rhodococci used in our previous experiments (Dhandapani et al., 2017, 2018). We also assessed the cytokinin content of peas inoculated with several different strains of *R. fascians* previously classified as avirulent or virulent (Stange et al., 1996), as well as peas inoculated with the closely related *Rhodococcus* sp. Leaf225 and *Williamsia* sp. Leaf354. *Rhodococcus* sp. Leaf225 and *Williamsia* sp. Leaf354 were isolated from healthy Arabidopsis grown under environmental conditions (Bai et al., 2015). *Williamsia* spp. are characterized by an unusual mycolic acid composition in their cell wall which places the genus between *Rhodococcus* and *Gordonia*. Interestingly, *Williamsia* infections in humans are often misdiagnosed as *Rhodococcus* infections (Keikha, 2018). Here we provide the first evidence that *Williamsia* spp. may cause fasciation in pea.

## Results

### Cytokinin Profiling of Inoculated Pea Tissue

As cotyledons, roots and shoots all show marked morphological responses to inoculation by virulent strains of *R. fascians*, the three tissues were analyzed separately from 5 days post-inoculation (dpi) to 35 dpi, while germinated cotyledons presenting a small root and shoot tissue were analyzed as the combined tissue at 2 dpi, and cotyledons alone at 4 h post-inoculation (hpi) as described in Dhandapani et al. (2017) and Dhandapani et al. (2018). At all times, mock-inoculated control samples were harvested alongside those seeds inoculated with the virulent or avirulent strain.

Previously, *t*Z, *t*ZR, *t*ZRMP, *t*ZOG, *t*ZROG, iP, iPR, iPRMP were reported in pea cotyledons, shoots and roots inoculated with virulent strain 602 and avirulent strain 589 as well as in mock-inoculated controls. There was no group of cytokinins or any specific cytokinin that correlated with virulence across all tissues (Dhandapani et al., 2017, 2018).

Continuing the analyses of the above plant material, we now show that the quantity of *c*Z in the *R. fascians* inoculated cotyledons, shoots and roots over the time course of the experiment generally exceeded those in the mock-inoculated controls (Table 1; Supplementary Table 1). However, frequently the amount of *c*Z was greater when the seeds had been inoculated with avirulent strain 589 (avir 589) than with virulent strain 602 (vir 602). Similarly for *c*ZR-5′-monophosphate (*c*ZRMP), the amounts were frequently greater than controls when seeds had been inoculated with the avirulent strain. There were no consistent patterns in the *c*Z-*O*-glucosides.

**Table 1:** *cis*-cytokinin content in *Pisum sativum* cotyledons, shoots and roots following either mock inoculation of seeds (CON), or inoculation with avirulent strain 589 (AVIR) or virulent strain 602 (VIR) of *Rhodococcus fascians*.

**Cytokinin(pmol/g DW)**	**TIME POINTS**																	
	**4 hpi**			**2 dpi**			**5 dpi**			**11 dpi**			**15 dpi**			**25 dpi**		
	**CON**	**AVIR**	**VIR**	**CON**	**AVIR**	**VIR**	**CON**	**AVIR**	**VIR**	**CON**	**AVIR**	**VIR**	**CON**	**AVIR**	**VIR**	**CON**	**AVIR**	**VIR**
**COTYLEDONS**																		
Total cZ	1.05	**0.4^b^**	**0.52**	9.16	7.62	8.3	19.9	25.3	17.1	48.6	**70.15**	**11.6**	36.4	41.95	34	17.9	**26.6**	15
cZ	0.16	0.17	**0.24**	0.15	**0.84**	**0.43**	0.19	**1.32**	**0.58**	1.01	**4.41**	0.86	0.62	**1.04**	0.7	0.68	**3.4**	**1.44**
cZR	0.89	**0.24**	**0.28**	0.65	0.74	0.64	0.77	**1.39**	0.97	1.57	**2.09**	**0.39**	2.66	2.52	2.97	1.19	**3.77**	1.55
cZRMP	<LOD^a^	<LOD	<LOD	10.4	**7.42**	**7.09**	17.3	20.9	13.4	43	51.52	**7.91**	15.1	**33.01**	**25.9**	6.34	**10.28**	5.2
cZOG	<LOD	<LOD	<LOD	1.26	1.52	1.25	0.98	0.91	**1.35**	1.76	**8.63**	**1.19**	9.63	**2.96**	**1.63**	4.45	4.06	**2.23**
cZROG	<LOD	<LOD	<LOD	0.74	**0.32**	0.52	0.66	0.76	0.74	1.29	**3.5**	1.21	8.39	**2.42**	**2.81**	6.22	5.1	4.57
cZ9G	<LOD	<LOD	<LOD	<LOD	<LOD	<LOD	<LOD	<LOD	<LOD	<LOD	<LOD	<LOD	<LOD	<LOD	<LOD	<LOD	<LOD	<LOD
**SHOOT**																		
Total cZ							150	190	**73.8**	40	**62.13**	**25.9**	28.5	**40.99**	27.9	17.3	**20.51**	**27.2**
cZ							2.31	**4.05**	**1.63**	0.69	**1.4**	**1.61**	0.76	**2.5**	**1.04**	0.91	1.04	0.85
cZR							12.4	**5.37**	**2.95**	1.97	**2.97**	**1.23**	3.02	**2.23**	2.92	1.47	1.3	**3.81**
cZRMP							121	**173**	**59.8**	31	**48.07**	**21.8**	17.8	**29.79**	17.3	9.23	**11.98**	**14.7**
cZOG							2.54	2.27	**3.67**	2.52	**5.02**	3.36	2.93	3.16	2.54	3.15	3.27	2.65
cZROG							12.3	**5.97**	**5.82**	3.84	4.67	3.82	4.05	3.32	4.07	2.58	2.92	**5.22**
cZ9G							<LOD	<LOD	<LOD	<LOD	<LOD	<LOD	<LOD	<LOD	<LOD	<LOD	<LOD	<LOD
**ROOT**																		
Total cZ							71.8	**108**	66.5	73.9	67.64	**37.4**	46.2	**81.49**	**60.9**	63.4	63.89	**48.1**
cZ							2.1	**5.94**	**3.23**	1.25	**3.93**	1.85	1.2	**6.3**	**7.2**	5.46	6.34	**1.24**
cZR							5.27	**7.87**	5.02	3.59	2.67	**1.44**	5.5	5.57	4.46	4.51	5.26	**3.08**
cZRMP							48.3	**79.4**	40.8	55.8	44.05	**24.2**	24.5	**50.85**	**34.9**	20	19.83	19.3
cZOG							2.83	**4.11**	**3.58**	3.54	**5.7**	**2.11**	2.24	**4.02**	**3.19**	3.77	3.93	**2.59**
cZROG							13	10.2	13.2	9.48	11.17	7.76	12.5	14.44	10.4	28.9	27.8	21.5
cZ9G							0.34	**0.83**	**0.61**	0.24	**0.15**	**0.04**	0.34	0.32	**0.65**	0.81	0.73	**0.39**

a *LOD-below detection limit*.

b *Each value is the average of four biological replicates, two from each of the two experiments. Bold numbers indicate values that are significantly different from control p <0.05*.

*Cotyledons were sampled 4 h post-inoculation (4 hpi) and 2 days post-inoculation (2 dpi), and cotyledons, shoots and roots at 5, 11, 15, and 25 dpi*.

No 2MeS-*t*Z or 2MeS-*t*ZR were detected in cotyledons, shoots or roots in peas at any stage. The amount of 2MeS-*c*Z in shoots inoculated with avir 589 (avir-shoots) was consistently greater than the mock controls, while for those inoculated with vir 602 (vir-shoots) 2MeS-*c*Z was not greater than that in avir-shoots (Table 2; Supplementary Table 1). Generally, 2MeS-*c*Z was greater than controls only in avir-roots. The amounts of 2MeS-*c*Z in cotyledons were generally very low until 25 dpi, when cotyledons inoculated with either avir 589 (avir- cots) or vir 602 (vir-cots) had similar levels that were greater than the controls. There was no consistent trend regarding the amounts of 2MeS-*c*ZR. At 15 dpi, when symptoms were most evident, the levels of 2MeS-*c*ZR were much less than the mock-inoculated controls in both vir- and avir- roots and shoots. The levels in vir-cots never exceeded those of the controls. Levels of 2MeS-iP were elevated in both vir-shoots and roots at 5 dpi, and not detectable in control or avir-roots or shoots. The levels in vir-cots never exceeded those of the controls. There was no consistent trend for 2MeS-iPR.

**Table 2:** 2-Methylthio-cytokinin content in *Pisum sativum* cotyledons, shoots and roots following either mock inoculation of seeds (CON), or inoculation with avirulent strain 589 (AVIR) or virulent strain 602 (VIR) of *Rhodococcus fascians*.

**Cytokinin(pmol/g DW)**	**TIME**																	
	**4 hpi**			**2 dpi**			**5 dpi**			**11 dpi**			**15 dpi**			**25 dpi**		
	**CON**	**AVIR**	**VIR**	**CON**	**AVIR**	**VIR**	**CON**	**AVIR**	**VIR**	**CON**	**AVIR**	**VIR**	**CON**	**AVIR**	**VIR**	**CON**	**AVIR**	**VIR**
**COTYLEDONS**																		
Total 2MeS-CK	0.023	**0.138^b^**	0.075	0.079	0.152	0.066	0.037	0.094	0.09	0.04	**0.221**	0.044	0.589	**0.177**	**0.333**	0.289	**0.793**	**0.593**
2MeS-iP	<LOD^a^	<LOD	<LOD	<LOD	<LOD	<LOD	<LOD	<LOD	<LOD	<LOD	<LOD	<LOD	0.514	<LOD	**0.297**	0.216	0.248	<LOD
2MeS-iPR	<LOD	<LOD	<LOD	<LOD	<LOD	<LOD	<LOD	<LOD	<LOD	<LOD	<LOD	<LOD	<LOD	<LOD	<LOD	0.099	**0.225**	**0.241**
2MeS-cZ	<LOD	0.099	0.063	<LOD	0.126	0.064	<LOD	<LOD	0.09	<LOD	<LOD	<LOD	<LOD	**0.089**	<LOD	0.049	**0.304**	**0.229**
2MeS-cZR	0.009	0.016	0.009	0.017	0.017	0.014	0.018	0.02	0.03	0.04	**0.158**	0.034	0.077	0.077	**0.048**	0.067	0.079	0.072
**SHOOT**																		
Total 2MeS-CK							0.421	0.388	**0.88**	0.506	**1.21**	0.507	1.056	0.883	**0.443**	1	1.099	0.858
2MeS-iP							<LOD	<LOD	0.56	<LOD	<LOD	<LOD	0.364	<LOD	0.337	0.263	0.439	<LOD
2MeS-iPR							0.087	<LOD	0.12	0.056	**0.162**	0.047	<LOD	0.125	0.121	0.155	0.227	**0.474**
2MeS-cZ							0.043	**0.2**	**0.12**	0.087	**0.167**	**0.177**	0.096	**0.176**	0.056	0.089	0.076	<LOD
2MeS-cZR							0.298	**0.153**	0.25	0.4	**1.043**	0.336	0.796	**0.586**	**0.157**	0.538	0.47	**0.371**
**ROOT**																		
Total 2MeS-CK							0.342	**0.544**	0.56	0.525	0.648	0.305	0.395	**0.597**	0.551	0.74	0.635	0.612
2MeS-iP							<LOD	<LOD	**0.46**	<LOD	<LOD	<LOD	<LOD	0.389	0.314	0.34	**0.198**	<LOD
2MeS-iPR							0.097	<LOD	**0.06**	0.088	**0.043**	**0.051**	<LOD	<LOD	0.88	0.285	**0.173**	0.346
2MeS-cZ							0.186	**0.404**	0.12	0.129	**0.426**	<LOD	0.067	**0.106**	**0.14**	0.063	**0.141**	0.078
2MeS-cZR							0.12	0.093	**0.18**	0.41	0.379	**0.196**	0.363	**0.09**	**0.092**	0.092	**0.123**	0.197

a *LOD-below detection limit*.

b *Each value is the average of four biological replicates, two from each of the two independent experiments. Bold numbers indicate values that are significantly different from control p < 0.05*.

*Cotyledons were sampled 4 h post-inoculation (4 hpi) and 2 days post-inoculation (2 dpi), and cotyledons, shoots and roots at 5, 11, 15, and 25 dpi*.

**Figure 1:**
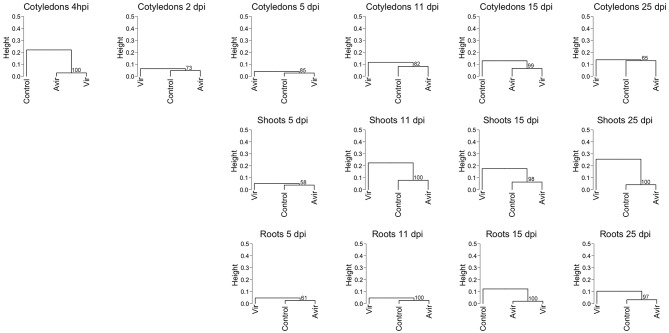
Hierarchical clustering of cytokinin content in pea cotyledons, shoots and roots inoculated with avirulent *R. fascians* strain 589, virulent strain 602 and mock inoculated control. Cluster analysis is based on cytokinin type and level detected in cotyledons at 4 h post-inoculation, and 2 days post-inoculation (dpi), and in cotyledons, shoots and roots from 5 to 25 dpi from four biological replicates. Dendrograms were constructed using Pearson correlation coefficients for the distance matrix and average linkage as the clustering method of log-transformed data, with bootstrapping of 1,000 iterations. Height represents the distance between nodes—the greater the height the greater the dissimilarity between treatments. Numbers in red indicate the unbiased approximate *p*-values (%), computed by multiscale bootstrap resampling using the *pvclust* R package (Suzuki and Shimodaira, 2006).

Recognizing the dynamic interconversions of the cytokinins, we constructed a hierarchical clustering of all cytokinin types against mock inoculated control and the two *R. fascians* strains, across all tissues and time points for all detected cytokinins (Figure 1). The clustering shows that, for example, in cotyledons, shoots and roots at 5 dpi there is little difference between the control and inoculated tissues, even though symptoms at this points are apparent (Dhandapani et al., 2017). At later time points the cluster analysis for shoots does indicate a difference between the cytokinins in vir-shoots compared to avir-shoots and mock-inoculated control, but the clustering is not consistent in cotyledons or roots. Although it cannot be ruled out that different tissue-specific homeostatic mechanisms are in part at the basis of this observation, it might also indicate that none of the cytokinins included in this analysis, nor their relative abundance to each other, is responsible for the symptoms across the tissues. Heat maps associated with the data, and which list all the included cytokinins, are shown in Supplementary Figure 1.

### *mt1* and *mt2* Genes in Strains of *Rhodococcus*

Both *mt1* and *mt2*, as well as fas4, were detected by PCR in all strains previously classed as virulent, as well as in *Rhodococcus* sp. Leaf225 and *Williamsia* sp. Leaf354 isolated from non-symptomatic Arabidopsis leaves (Supplementary Table 2).

### Expression of *mt1* and *mt2*

**Figure 2:**
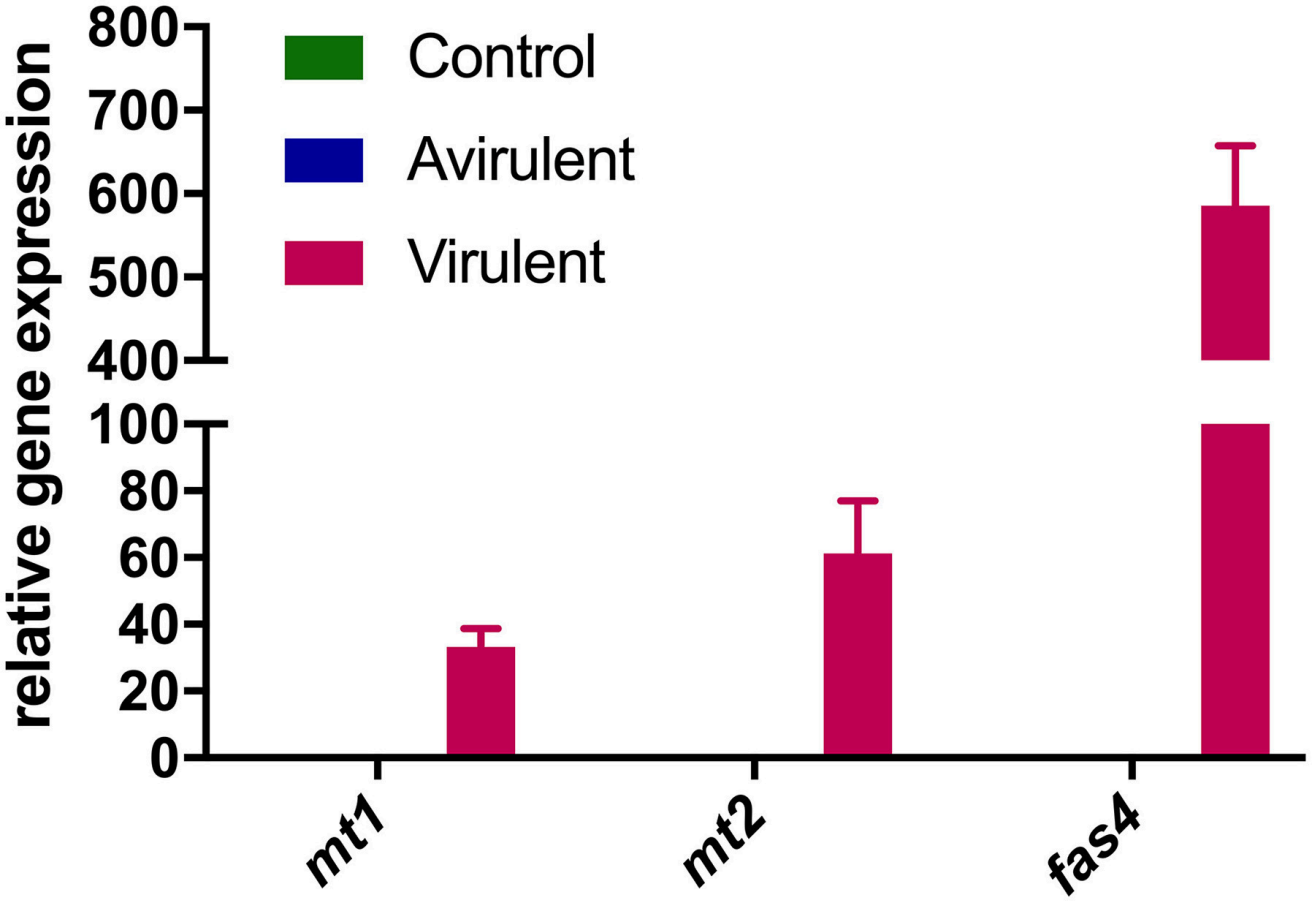
Relative expression of *mt1, mt2*, and *fas4* in *R. fascians* cultures. Expression was determined following culture of *R. fascians* avirulent strain 589 (Avirulent) and virulent strain 602 (Virulent), followed by RNA extraction and cDNA synthesis. A no-template reaction was used as control. Relative fold changes were determined using *16S rRNA* as internal reference gene. The error bars are ± 1 s.d. of the three biological replicates in the RT-qPCR.

**Figure 3:**
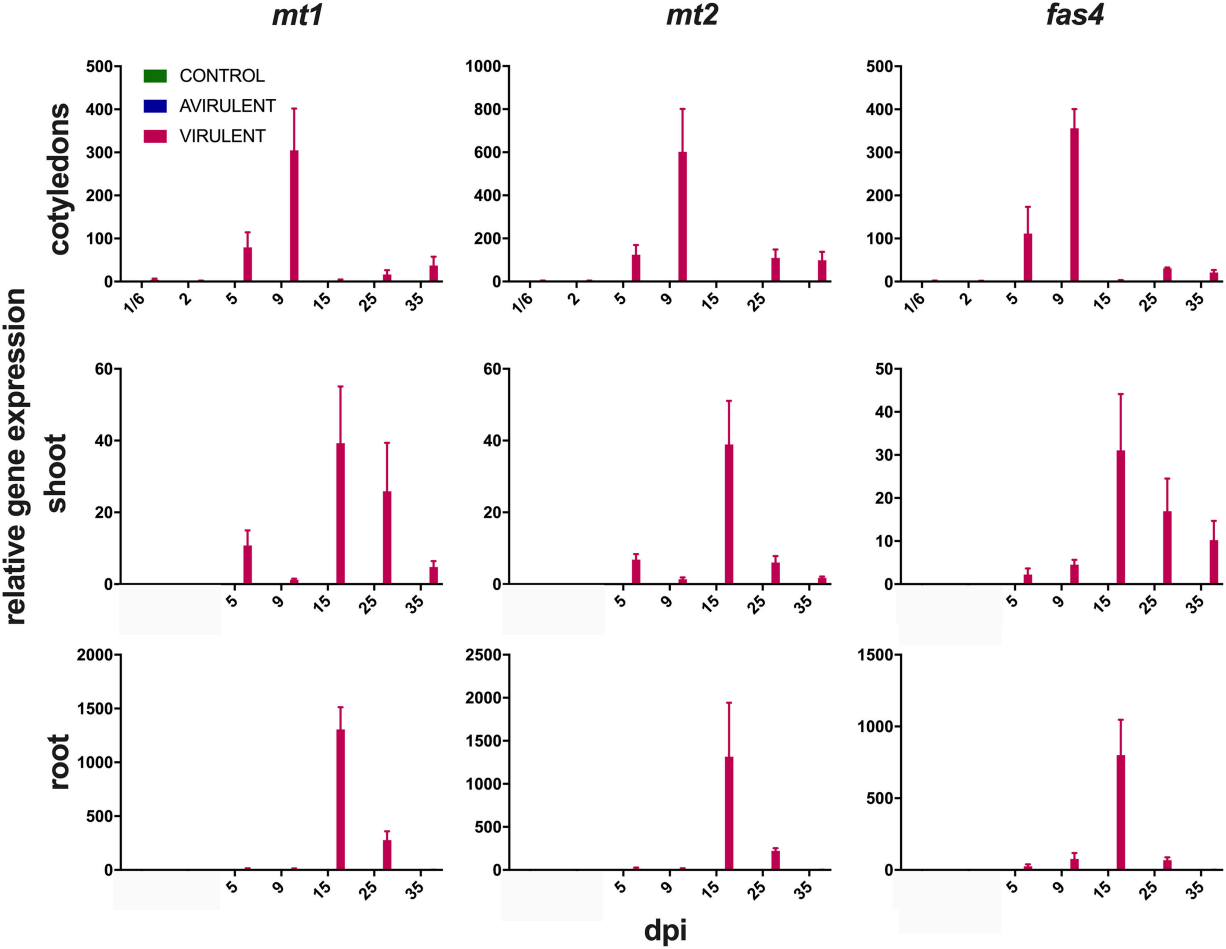
Expression of *mt1, mt2*, and *fas4* in cotyledons from 4 h post-inoculation (^1^/_6_ days post-inoculation; dpi) to 35 dpi, and in shoots and roots from 5 to 35 dpi following seed inoculation with either virulent *R. fascians* strain 602, avirulent strain 589, or mock inoculation. Relative fold changes were calculated using *GAPDH* and *18S rRNA* as internal reference genes. The error bars are ± 1 s.d. of two independent experiments.

Strong expression of *mt1, mt2*, and *fas4* were detected in *in vitro* cultures of vir 602 (Figure 2). The expression of *mt1* and *mt2* was the strongest in germinating cotyledons 9 dpi, and in shoots and roots at 15 dpi, and in a similar pattern to *fas4* in those plants inoculated with virulent strain 602 (Figure 3).

### Detection of 1-MeiP and 2-MeiP

**Figure 4:**
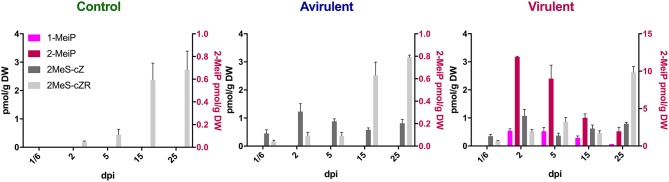
Content of four cytokinins (pmol/g DW) in *Pisum sativum* cotyledons from 4 h post-inoculation (^1^/_6_ days post-inoculation; dpi) to 25 dpi following mock inoculation of pea seeds (Control), or inoculation with *R. fascians* strains 589 (Avirulent) or 602 (Virulent). The error bars are ± s.d. of four biological replicates.

**Figure 5:**
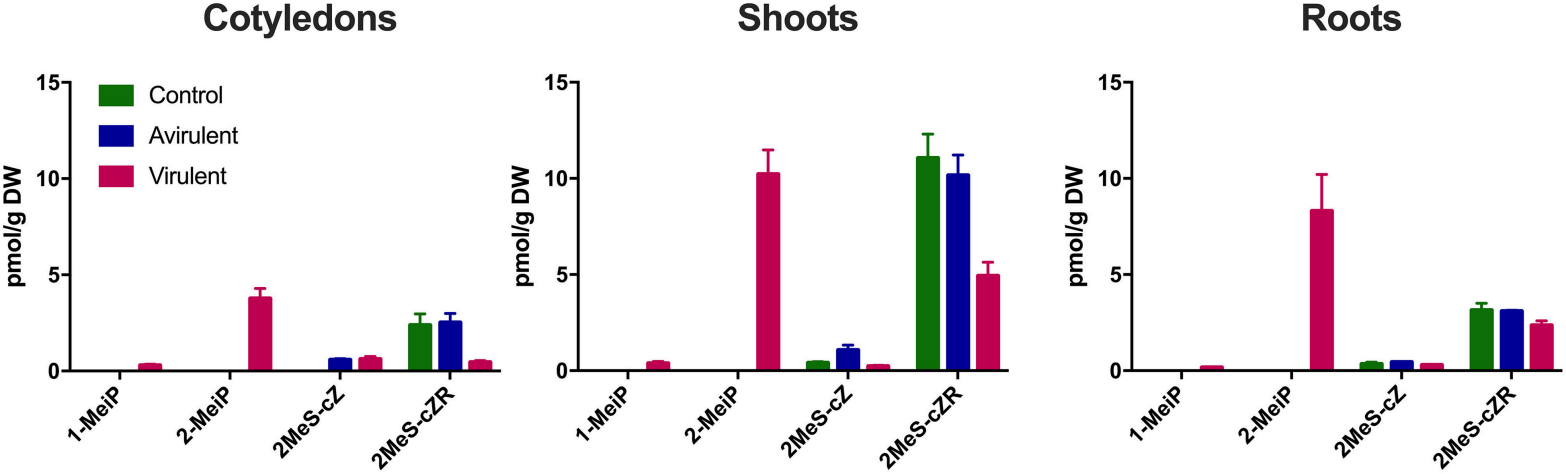
Comparison of four cytokinins (pmol/g DW) in cotyledons, shoots and roots 15 days post-inoculation with *R. fascians* virulent strain 602, avirulent strain 589, or mock inoculation (Control). The error bars are ± s.d. of four biological replicates.

Both 1-MeiP and 2-MeiP were detected in cotyledons inoculated with vir 602 from 2 to 25 dpi, but not at 4 hpi (Figure 4). The quantity of 2-MeiP was generally an order of magnitude > 1-MeiP. Shoot and root tissues were analyzed at 15 dpi, and both methylated cytokinins were detected. No methylated cytokinin was detected in tissues inoculated with avir 589 or mock-inoculated control tissues, but both 2MeS-*c*Z and 2MeS-*c*ZR were detected in all three samples (Figure 5).

### Cytokinin Analysis of Peas Inoculated With Different Bacterial Strains

Both the *Rhodococcus* Leaf225 and *Williamsia* Leaf354 strains caused strong fasciation symptoms on pea (Supplementary Figure 2). Twenty-five different cytokinins were detected in the cotyledons, shoot and roots of inoculated pea plants (Supplementary Table 3). 1-MeiP was detected in cotyledons, shoots and roots at 15 days after inoculation with Vir1, and at higher levels in shoots 15 dpi with Vir4 and Vir5. These tissues were not analyzed for 2-MeiP, as the standard was not available at the time.

**Figure 6:**
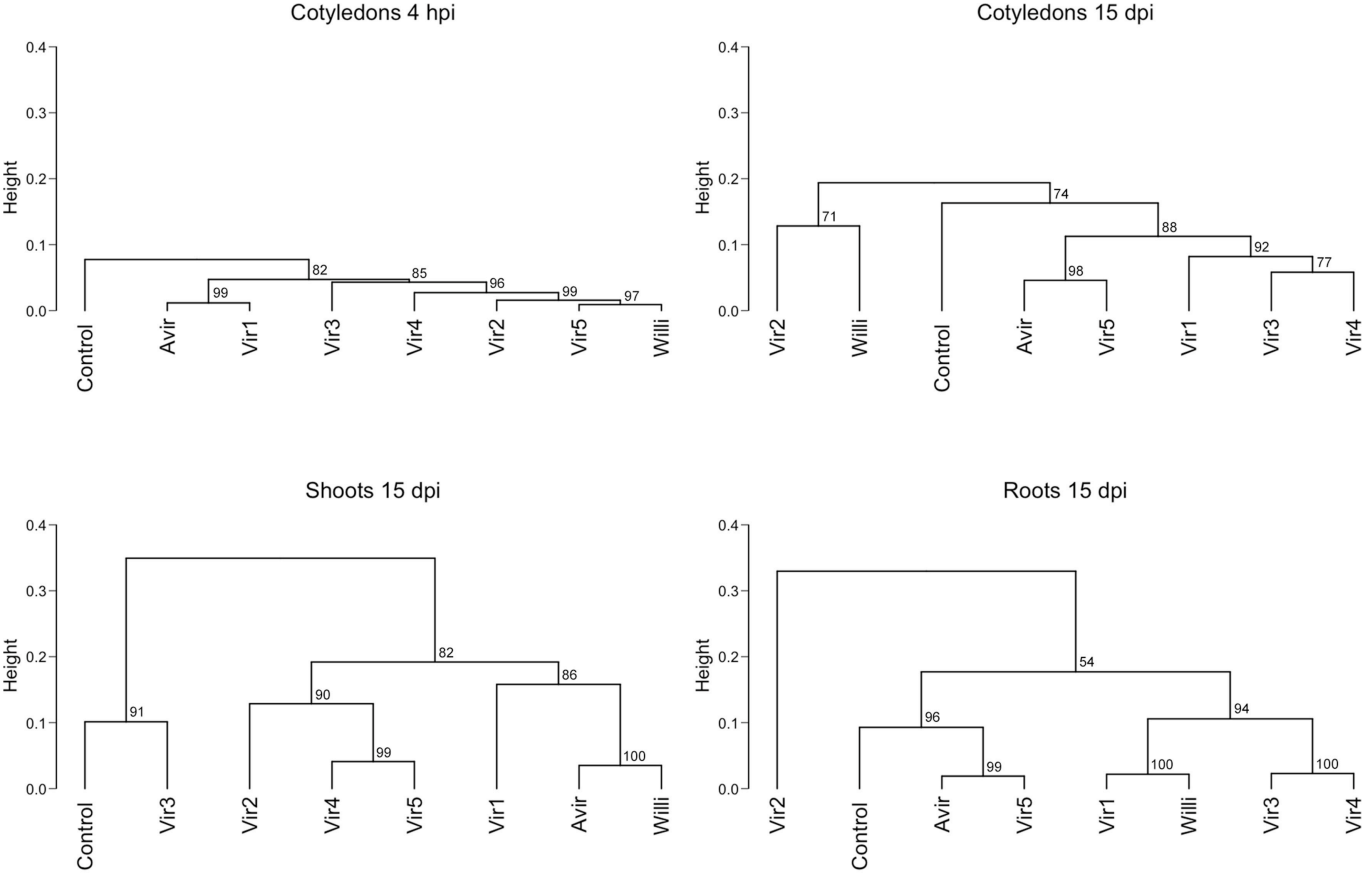
Hierarchical clustering of cytokinin content in pea cotyledons, shoots and roots inoculated with different bacterial strains. Cluster analysis is based on cytokinin type and level detected in cotyledons at 4 h post-inoculation (hpi), and in cotyledons, shoots and roots at 15 days post-inoculation (dpi) with an avirulent strain, six virulent strains, and mock-inoculated control, from four biological replicates. Dendrograms were constructed using Pearson correlation coefficients for the distance matrix and average linkage as the clustering method of log-transformed data, with bootstrapping of 1,000 iterations. Height represents the distance between nodes—the greater the height the greater the dissimilarity between treatments. Numbers in red indicate the unbiased approximate *p*-values (%), computed by multiscale bootstrap resampling using the *pvclust* R package (Suzuki and Shimodaira, 2006). Strains are as described in Stange et al. (1996): Avirulent—avirulent strain 593; Vir 1: virulent strain 594 (210 kb linear plasmid similar to virulent strain 602); Vir 2: 599 (same RFLP pattern to 602 but linear plasmid not detected); Vir 3: 666 (has a circular plasmid); Vir 4: 606 (130 kb linear plasmid). Vir5: *R. fascians* sp. Leaf225 isolated from non-symptomatic Arabidopsis leaves and Willi: *Williamsia* sp. Leaf354, also isolated from non-symptomatic Arabidopsis leaves.

A hierarchical clustering of cytokinin type against mock-inoculated control, the avirulent strain and six virulent strains in cotyledons at 4 hpi, and in cotyledons, shoots and roots at 15 dpi, showed that there was no consistent pattern linking cytokinin abundances to virulent strains (Figure 6; Supplementary Figure 3). High dissimilarity was detected across all samples at 15 dpi. Mock controls and avirulent strains clustered randomly between virulent strains.

## Discussion

It is well-known that both virulent and avirulent strains of *R. fascians* secrete a similar array of cytokinins into culture medium (e.g., Scarbrough et al., 1973; Armstrong et al., 1976; Murai et al., 1980; Eason et al., 1996; Pertry et al., 2009). Consequently, we provide comparisons at all times between mock inoculated seeds and seeds inoculated with both virulent and, importantly, avirulent strains, thus providing a true comparison and analysis of the critical differences induced by virulent strains, which cause the fasciation syndrome.

Pertry et al. (2009) showed transient increases of iP, 2MeS-iP, and 2MeS-*t*Z, and more stable increased levels of *c*Z and 2MeS-*c*Z, relative to mock-inoculated controls, in Arabidopsis infected with virulent strain D188, but did not show the comparison with D188-5—the plasmid-free avirulent strain. However, neither the 2MeS-CK derivatives nor *c*Z accumulated in infected tobacco tissues while iP did (Pertry et al., 2009; Radhika et al., 2015). In contrast, in inoculated pea, both methylthio-derivatives and iP were detected. However, we showed that there was no correlation between the levels of the 2MeS-*c*Z and 2MeS-*c*ZR, or 2MeS-iP and 2MeS-iPR and virulence in pea. Likewise, for the *c*Z-type cytokinins. Creason et al. (2014) also reported that *c*Z and 2MeS-*c*Z accumulated independently of *fas* gene induction in cultures. All these cytokinins could derive from tRNA (Murai et al., 1980; Pertry et al., 2010; Francis et al., 2016). Based on the above cluster analyses (Figures 1, 6) we suggest that the “Trick-with-the-Cytokinin-Mix” hypothesis of Pertry et al. (2010) does not hold in terms of generality to plants considered host species for *R. fascians*, as foreshadowed in Francis and Vereecke (2019) who recently stated “time will tell if the *fas* operon and the Trick-with-the-Cytokinin-Mix are indeed determinative for leafy gall formation.”

However, the hypothesis promoted by Creason et al. (2014), who suggested that the iP-type cytokinins alone can account for the morphogenetic abnormalities, does not hold either. Although increased levels of iP are shown in infected tobacco tissues relative to mock inoculated controls (Pertry et al., 2009; Radhika et al., 2015), there was no correlation with virulence in peas based on the analysis of iP-type cytokinins in cotyledons (Dhandapani et al., 2017) and roots and shoots (Gális et al., 2005; Dhandapani et al., 2018). Additionally, when several different *R. fascians* strains were assessed against an avirulent strain and relative to mock-inoculated controls, there was considerable variation between strains with respect to the standard cytokinins. As suggested previously by Jameson (2000), the iP-type cytokinins are probably unrelated to virulence.

Subsequently, based on the identification of the mono- and dimethylated cytokinins (1-MeiP and 2-MeiP, respectively) in tobacco (Radhika et al., 2015), we sought to establish the presence of the *mt1* and *mt2* genes in a set of *Rhodococcus* strains and their expression in inoculated pea plants. There is clear evidence that both the *mt1* and *mt2* genes are present in all *R. fascians* strains classed as virulent, and are absent in those strains classed as avirulent, and there is clear evidence for expression of both genes in plants inoculated with the virulent strain 602. There is also a similar pattern of expression of *Fas4/D*—the gene that codes for the enzyme which attaches the double methylated side chain to the adenine moiety, and which was previously described as an isopentenyl transferase.

To further establish a role for the methylated cytokinins, 1-MeiP and, subsequently, 2-MeiP, were synthesized and quantified in cotyledons, shoots and roots of pea inoculated with virulent and avirulent strains of *Rhodococcus*. Initially (when only 1-MeiP was available), we detected low levels of 1-MeiP in extracts of peas previously inoculated with Vir1, Vir4, or Vir5. These data are similar to those reported for tobacco where 1-MeiP was only detected at low levels in leaf extracts of inoculated tobacco (Radhika et al., 2015). 1-MeiP in the other strains may have been below the limit of detection.

In tobacco, the levels of 2-MeiP were much greater and similar to those of the iP-type cytokinins (Radhika et al., 2015). Our quantitative data also showed that the 2-MeiP levels are comparable to the published endogenous iP content in pea cotyledons, shoots and roots following seed inoculation with *R. fascians* (Dhandapani et al., 2017, 2018). We could not detect 1- or 2-MeiP in peas inoculated with the avirulent strain nor by the mock-inoculated control.

As Radhika et al. (2015) highlighted, methylation of key compounds has been used by invading organisms to bypass detection/challenge by the plant system. Interestingly, different methylated cytokinins are produced in culture by certain strains of the gall-inducing bacterium *Pseudomonas syringae* pv. *savastanoi* (Evidente et al., 1986, 1991; MacDonald et al., 1986). In this case, the methylation of *t*Z and *t*ZR occurred at a different position on the side chain (the 1″ position). Substantial quantities of these cytokinins, along with significant quantities of *t*Z and *t*ZR, were identified from culture medium but no evaluation of these cytokinins *in planta* appears to have been carried out.

It is interesting to note that the additionally tested strains, *Rhodococcus* sp. Leaf225 and *Williamsia* sp. Leaf354, which exhibited pathogenic fasciation on pea, were isolated from asymptomatic Arabidopsis plants. This indicates that in complex environmental communities these Corynebacteriaceae may exist as non-pathogenic epiphytes. As highlighted by Stes et al. (2013), the pathogenic behavior may be population density related. However, we did not observe disease symptoms on gnotobiotic Arabidopsis inoculated with these strains (Schlechter and Remus-Emsermann, unpublished), in contrast to the marked disease symptoms on pea, supporting the continued use of pea as a model system for studying fasciation-inducing bacteria.

In conclusion, based on the information that the *mt1* and *mt2* mutants of *R. fascians* are non-pathogenic (Pertry, 2009), resistance of the 2-MeiP to CKX (Radhika et al., 2015), the susceptibility of the iP-type cytokinins to CKX (Pertry et al., 2009; Radhika et al., 2015), and the lack of consistency of iP-type levels between tissue types and between virulent strains [(Dhandapani et al., 2017, 2018); this work], we suggest the most likely cause of the symptoms expressed in pea is primarily due to the production of the novel methylated cytokinins, with the methylation enhancing the movement by diffusion of cytokinin through plant membranes as proposed by Radhika et al. (2015). The 1- and 2-MeiP cytokinins are the only cytokinins so far identified that are uniquely produced by the virulent *R. fascians* strains, although the possibility exists that they may also be found in galls formed by *A. tumefaciens, P. s*. pv*. savastanoi* or *Pantoa agglomerans* pv. *gypsophilae*. Further work is needed investigating multiple strains of *R. fascians* on multiple host plants to assess the roles of the methylated cytokinins, the cytokinins derived from tRNA turnover and the genes of the *fas* operon both spatially and temporally during the development of the leafy gall syndrome.

## Materials and Methods

### Plant Material and Bacterial Strains

The origin of 36 strains of *Rhodococcus fascians* is listed in Supplementary Table 4. *Rhodococcus* sp. Leaf225 and *Williamsia* sp. Leaf354 were isolated from non-symptomatic Arabidopsis leaves (Bai et al., 2015).

*Pisum sativum* var. Bohatyr seeds were inoculated with *R. fascians* virulent strain 602 [vir 602; containing the linear virulence plasmid (Stange et al., 1996)] and the avirulent strain 589 (avir 589; lacking the virulence plasmid). The plants were sampled over time and used for the analyses of the *c*Z-types and the 2MeS cytokinins, and for 1-MeiP and 2-MeiP, as well as the *in planta* expression analyses of *mt1, mt2*, and *fas4*. These samples were obtained from the same experimental material as previously used for the analyses described in detail in Dhandapani et al. (2017) and (Dhandapani et al., 2018).

### Identification of *mt1* and *mt2* in *Rhodococcus fascians* Strains

Bacterial cultures (four plates per strain) were cultivated on Kado and Heskett “523” agar medium (Kado and Heskett, 1970) and grown at 26°C for 2 days. To obtain DNA for PCR, a modified method from Gális et al. (2005) was used. A sterile toothpick was dipped into bacterial colonies and a small portion was subsequently transferred into 20 μl of sterile water. The culture was mixed by vortexing and 1 μl of suspension was used for PCR.

The *R. fascians* S-adenosyl methionine-dependent methyltransferases (*mt1* and *mt2*), adenosine phosphate-isopentenyl transferase (*fas4*), *dprA* and *16S rRNA* were identified using the NCBI GenBank database. Specific primers were designed, using Primer Premier TM 5.00, to anneal to *R. fascians* and not to pea genomic DNA. The primers designed and used for the PCR reactions are listed in Supplementary Table 5. The sequence for *dprA* is located on the linear plasmid pFiD188 of *R. fascians* strain D188. The PCR conditions for amplification of the genes are outlined in Dhandapani et al. (2017). The annealing temperature used for all genes was 55°C. All 36 isolates of *R. fascians*, along with *Rhodococcus* sp. Leaf225 and *Williamsia* sp. Leaf354 were screened for the presence/absence of *mt1, mt2, fas4, dprA*, and *16S rRNA* genes. The PCR was repeated 4 times for each plate.

### Expression of *mt1, mt2*, and *fas4* in *R. fascians* Broth Cultures

Expression of *mt1, mt2*, and *fas4* in broth cultures of *R. fascians* vir 602 and avir 589 was determined using RT-qPCR. Bacterial cultures (three flasks for each strain) were grown over night in “523” broth (Kado and Heskett, 1970) at 26°C with shaking at 150 rpm. Mid-log phase cultures were then inoculated into Klämbt broth (Klämbt et al., 1966), incubated for 4 h at 26°C with shaking at 150 rpm. The cultures were then harvested by centrifugation at 15,000 rpm for 15 min at 4°C. The supernatant was discarded and the pellets were re-suspended in TRIzol^®^ reagent (Invitrogen, Carlsbad, CA, USA). RNA extraction and cDNA synthesis were performed as described earlier (Dhandapani et al., 2017). Four cDNAs were produced from each flask. These cDNAs were then pooled to provide one biological replicate. RT-qPCR was performed for the genes *mt1, mt2*, and *fas4*, with *16S rRNA* used as a reference gene, using KAPA SYBR^®^ FAST qPCR kits (Kapa Biosystems, Boston, USA) in a Qiagen Rotor-Gene Q (Dhandapani et al., 2017, 2018). Primer sequences are listed in Supplementary Table 5. Three technical replicates were run for each of the pooled cDNAs.

### Expression of *mt1, mt2*, and *fas4* in Inoculated Pea Tissues

RT-qPCR was used to evaluate the relative expression of *mt1, mt2*, and *fas4* genes in cotyledons, shoots and roots at various time intervals following inoculation of Bohatyr seeds with *R. fascians* vir 602, avir 589 and the mock inoculated control as described in Dhandapani et al. (2017) and Dhandapani et al. (2018). The primers used for the RT-qPCR analysis are listed in Supplementary Table 5. Expression analysis of the target genes, *mt1, mt2*, and *fas4*, followed the RT-qPCR procedure outlined in Dhandapani et al. (2017) and adhered to the MIQE guidelines (Bustin et al., 2009), with three technical replicates for each cDNA. The experiment was repeated twice as described in Dhandapani et al. (2017) and the averaged fold-change values presented for each time point and tissue. The reference genes *GAPDH* and *18S rRNA* were used as internal controls to normalize the data following the procedure used by Song et al. (2012) and Ninan et al. (2019). As the data derived from the two independent experiments were so consistent the experiment was not repeated a third time.

### Cytokinin Analysis

Naturally occurring and deuterium-labeled isoprenoid and 2-methylthio CK standards were obtained from Olchemim Ltd. (Czech Republic). Methylated cytokinins (1-MeiP and 2-MeiP) were synthesized by the procedure published previously by Radhika et al. (2015).

Four lyophilized biological replicates (3 mg of dry weight derived from four individual plants, two plants from each experiment) were extracted in 1 ml of modified Bieleski buffer (Hoyerová et al., 2006) together with a cocktail of stable isotope-labeled internal standards (0.2 pmol of cytokinin bases, ribosides, *N*-glucosides, and 2MeS-CK bases, ribosides, and 0.5 pmol of cytokinin *O*-glucosides, nucleotides per sample added). The samples were incubated at 4°C with continuous shaking (30 min), centrifuged (15 min, 23,000 *g* at 4°C) and then purified by a two-step solid phase extraction (SPE) method. All cytokinin metabolites (isoprenoid, 2-methylthio and methylated cytokinins) were isolated using a combination of silica-based (C_18_, 100 mg/mL; Applied Separations, Allentown, PA, USA) and mixed-mode (Oasis^®^ MCX, 30 mg/3 mL; Waters, Milford, MA, USA) sorbents (Dobrev and Kamínek, 2002). Analytes were eluted by three-step elution using a 0.35 M NH_4_OH aqueous solution and 0.35 M NH_4_OH in 80% (v/v) methanol solution. After SPE purification, eluates were evaporated to dryness and dissolved in 50 μl of 10% methanol prior to mass analysis using an Acquity UPLC^®^ I-Class System linked to a Xevo™ TQ-S MS spectrometer (Waters Corp., Milford, MA).

The purified samples (10 μL) were injected onto a C_18_ reversed-phase column (BEH Shield C_18_; 1.7 μm; 2.1 × 150 mm; Waters) and the isoprenoid cytokinins were determined according to the method described by Svačinová et al. (2012). The second half of the samples was injected onto a C_4_ reversed-phase column (Jupiter 5 μm C4, 150 × 2.0 mm; Phenomenex, Torrace, CA), and eluted with a linear gradient (0–10 min, 12% B; 12–18 min, 12–16% B; 18–23 min, 16–60% B; flow-rate of 0.4 mL/min; column temperature of 30°C) of 20 mM ammonium formate (pH 5.0, A) and acetonitrile (B). Quantification 2-methylthio-derivatives of isoprenoid cytokinin and methylated cytokinins was obtained by multiple reaction monitoring of [M+H]^+^ and the appropriate product ion as described previously by Tarkowski et al. (2010) and Radhika et al. (2015), respectively. All chromatograms were analyzed using MassLynx software version 4.1 (Waters), and the compounds were quantified by standard isotope dilution analysis.

### Cytokinin Analyses of Pea Tissues Inoculated With Additional *Rhodococcus* Strains

A new experiment was established using a temperature and humidity-controlled growth cabinet (CMP6010, Conviron, Winnipeg, Canada) after the original growth rooms were demolished with the demolition of the building following the Christchurch earthquakes. Pea seeds var. Onward [previously shown to be highly responsive to virulent strains of *R. fascians* (Eason et al., 1995; Stange et al., 1996)] were surface sterilized and inoculated with different strains. These strains are described in Stange et al. (1996): avir: avirulent strain 593; Vir1: virulent strain 594 (210 kb linear plasmid similar to virulent strain 602); Vir2: 599 (same RFLP pattern to vir 602 but linear plasmid not detected); Vir3: 666 (has a circular plasmid); Vir4: 606 (130 kb linear plasmid). Vir5: *Rhodococcus* sp. Leaf225 isolated from non-symptomatic Arabidopsis leaves and *Williamsia* sp. Leaf354, also isolated from non-symptomatic Arabidopsis leaves, were also included, along with mock-inoculated controls.

Five seeds from each treatment were placed in 500 ml containers with 0.6% (w/v) agar and 10% Hoagland's mineral salt solutions (Lawson et al., 1982). The containers were placed in a growth cabinet at 21°C with a 16 h photoperiod and 75% humidity. Samples of cotyledons 4 h post-inoculation (hpi) and separated cotyledons, shoots and roots at 15 days post inoculation (dpi) were collected and immediately immersed in liquid nitrogen and stored at −80°C for endogenous cytokinins analysis. For each sampling, tissues from four individual plants from independent inoculations were collected from each of the treatments to make four biological replicates. Tissues were then ground under liquid nitrogen and freeze-dried. All samples were extracted and purified as described above. Cytokinin levels were determined by an ultra-high performance liquid chromatography-electrospray tandem mass spectrometry (LC-MS/MS) using stable isotope-labeled internal standards as a reference (Rittenberg and Foster, 1940).

It is important to note that there is a discrepancy in the levels of the methylthio-cytokinins which were analyzed at the same time as the data published in Dhandapani et al. (2017, 2018) and presented in Table 2 and Supplementary Table 1, and that analyzed more recently (Figures 4, 5 and Supplementary Table 3). This is due to a different standard curve being used, and potentially partly due to long term storage of the samples and different degrees of lyophilisation of the samples. However, the relative ratios between mock-inoculated control and *R. fascians*-inoculated samples is similar (data not shown), and this discrepancy becomes irrelevant when the hierarchical clustering analysis is performed (Figures 1,6).

## Data Availability

All datasets generated for this study are included in the manuscript and/or the Supplementary Files.

## Author Contributions

PJ conceived and supervised the project. ON and MS provided all the cytokinin analyses. MZ synthesized the methylated cytokinins. JS co-supervised the project, analyzed the pea transcriptome, and developed the primers. PD conducted all experiments, the PCR and the RT-qPCR, and compiled the expression data. MR-E analyzed the bacterial strains from Arabidopsis leaves and with RS, developed and described the dendrogram analysis. PJ wrote the original draft of the paper with input from MR-E and PD. All authors contributed and approved the final manuscript.

### Conflict of Interest Statement

The authors declare that the research was conducted in the absence of any commercial or financial relationships that could be construed as a potential conflict of interest.
