# Supplementary material for: The Cytokinin Complex Associated With Rhodococcus fascians: Which Compounds Are Critical for Virulence?
Source: Front Plant Sci. 2019 May 22;10:674. doi: 10.3389/fpls.2019.00674 (PMC6539147; doi:10.3389/fpls.2019.00674)
Supplement: Supplementary file 1 [file Table_1.pdf]

Table - 2-MeS-cytokinin levels in pmol/g DW (Mean ± SD)

Asterisks indicate statistically significant difference in the pea plants inoculated with *Rhodococcus fascians* avirulent strain 589/virulent strain 602 versus the control in an ANOVA analysis (t-test; *, **, and *** correspond to P-values of 0.05 > p > 0.01, 0.01 > p > 0.001, and p < 0.001, respectively).

Timepoint	*Tissue*	*Status*	*Total 2MeS-CKs*	*2MeS-tZ*	*2MeS-tZR*	*2MeS-cZ*	*2MeS-cZR*	*2MeS-iP*	*2MeS-iPR*
4 hours post-inoculation	Cotyledon	*Control*	0.023 ± 0.016	*<LOD*	*<LOD*	*<LOD*	0.009 ± 0.000	*<LOD*	*<LOD*
		Avirulent	**0.138 ± 0.079** *	*<LOD*	*<LOD*	0.099 ± 0.031	0.016 ± 0.005	*<LOD*	*<LOD*
		Virulent	0.075 ± 0.063	*<LOD*	*<LOD*	0.063 ± 0.001	0.009 ± 0.003	*<LOD*	*<LOD*
2 days post-inoculation	Cotyledon	*Control*	0.079 ± 0.070	*<LOD*	*<LOD*	*<LOD*	0.017 ± 0.006	*<LOD*	*<LOD*
		Avirulent	0.152 ± 0.081	*<LOD*	*<LOD*	0.126 ± 0.032	0.017 ± 0.005	*<LOD*	*<LOD*
		Virulent	0.066 ± 0.033	*<LOD*	*<LOD*	0.064 ± 0.021	0.014 ± 0.004	*<LOD*	*<LOD*
5 days post-inoculation	Cotyledon	*Control*	0.037 ± 0.029	*<LOD*	*<LOD*	*<LOD*	0.018 ± 0.004	*<LOD*	*<LOD*
		Avirulent	0.094 ± 0.128	*<LOD*	*<LOD*	*<LOD*	0.023 ± 0.004	*<LOD*	*<LOD*
		Virulent	0.091 ± 0.018	*<LOD*	*<LOD*	0.087 ± 0.017	0.026 ± 0.007	*<LOD*	*<LOD*
	Shoot	*Control*	0.421 ± 0.054	*<LOD*	*<LOD*	0.043 ± 0.008	0.298 ± 0.031	*<LOD*	0.087 ± 0.017
		Avirulent	0.388 ± 0.028	*<LOD*	*<LOD*	**0.200 ± 0.029** ***	**0.153 ± 0.024** **	*<LOD*	*<LOD*
		Virulent	**0.875 ± 0.126** **	*<LOD*	*<LOD*	**0.119 ± 0.037** ***	0.254 ± 0.058	0.564 ± 0.245	0.118 ± 0.034
	Root	*Control*	0.342 ± 0.063	*<LOD*	*<LOD*	0.186 ± 0.049	0.120 ± 0.017	*<LOD*	0.097 ± 0.013
		Avirulent	**0.544 ± 0.098** *	*<LOD*	*<LOD*	**0.404 ± 0.042** ***	0.093 ± 0.024	*<LOD*	*<LOD*
		Virulent	0.557 ± 0.292	*<LOD*	*<LOD*	0.124 ± 0.008	**0.178 ± 0.020** **	**0.456 ± 0.144** ***	**0.055 ± 0.007** **
11 days post-inoculation	Cotyledon	*Control*	0.040 ± 0.003	*<LOD*	*<LOD*	*<LOD*	0.040 ± 0.003	*<LOD*	*<LOD*
		Avirulent	**0.221 ± 0.097** **	*<LOD*	*<LOD*	*<LOD*	**0.158 ± 0.013** ***	*<LOD*	*<LOD*
		Virulent	0.044 ± 0.019	*<LOD*	*<LOD*	*<LOD*	0.034 ± 0.004	*<LOD*	*<LOD*
	Shoot	*Control*	0.506 ± 0.074	*<LOD*	*<LOD*	0.087 ± 0.019	0.400 ± 0.033	*<LOD*	0.056 ± 0.009
		Avirulent	**1.210 ± 0.230** ***	*<LOD*	*<LOD*	**0.167 ± 0.018** **	**1.043 ± 0.214** ***	*<LOD*	**0.162 ± 0.010** ***
		Virulent	0.507 ± 0.121	*<LOD*	*<LOD*	**0.177 ± 0.046** **	0.336 ± 0.062	*<LOD*	0.047 ± 0.012
	Root	*Control*	0.525 ± 0.064	*<LOD*	*<LOD*	0.129 ± 0.030	0.410 ± 0.042	*<LOD*	0.088 ± 0.020
		Avirulent	0.648 ± 0.085	*<LOD*	*<LOD*	**0.426 ± 0.097** **	0.379 ± 0.124	*<LOD*	**0.043 ± 0.007** *
		Virulent	0.305 ± 0.130	*<LOD*	*<LOD*	*<LOD*	**0.196 ± 0.060** **	*<LOD*	**0.051 ± 0.013** *
15 days post-inoculation	Cotyledon	*Control*	0.589 ± 0.046	*<LOD*	*<LOD*	*<LOD*	0.077 ± 0.016	0.514 ± 0.062	*<LOD*
		Avirulent	**0.177 ± 0.060** ***	*<LOD*	*<LOD*	**0.089 ± 0.025** ***	0.077 ± 0.023	*<LOD*	*<LOD*
		Virulent	**0.333 ± 0.106** *	*<LOD*	*<LOD*	*<LOD*	**0.048 ± 0.007** *	**0.297 ± 0.075** *	*<LOD*
	Shoot	*Control*	1.056 ± 0.247	*<LOD*	*<LOD*	0.096 ± 0.030	0.796 ± 0.106	0.364 ± 0.042	*<LOD*
		Avirulent	0.883 ± 0.184	*<LOD*	*<LOD*	**0.176 ± 0.040** *	**0.586 ± 0.032** *	*<LOD*	0.125 ± 0.030
		Virulent	**0.443 ± 0.155** **	*<LOD*	*<LOD*	0.059 ± 0.002	**0.157 ± 0.040** ***	0.337 ± 0.097	0.121 ± 0.035
	Root	*Control*	0.395 ± 0.093	*<LOD*	*<LOD*	0.067 ± 0.001	0.363 ± 0.081	*<LOD*	*<LOD*
		Avirulent	**0.597 ± 0.083** *	*<LOD*	*<LOD*	**0.106 ± 0.008** *	**0.090 ± 0.018** ***	0.389 ± 0.063	*<LOD*
		Virulent	0.551 ± 0.052	*<LOD*	*<LOD*	**0.140 ± 0.027** *	**0.092 ± 0.013** ***	0.314 ± 0.032	0.088 ± 0.015
25 days post-inoculation	Cotyledon	*Control*	0.289 ± 0.075	*<LOD*	*<LOD*	0.049 ± 0.010	0.067 ± 0.013	0.216 ± 0.065	0.099 ± 0.014
		Avirulent	**0.793 ± 0.121** ***	*<LOD*	*<LOD*	**0.304 ± 0.040** ***	0.079 ± 0.018	0.248 ± 0.082	**0.225 ± 0.064** **
		Virulent	**0.593 ± 0.091** **	*<LOD*	*<LOD*	**0.229 ± 0.047** ***	0.072 ± 0.011	*<LOD*	**0.241 ± 0.077** **
	Shoot	*Control*	1.000 ± 0.136	*<LOD*	*<LOD*	0.089 ± 0.008	0.538 ± 0.117	0.263 ± 0.085	0.155 ± 0.032
		Avirulent	1.099 ± 0.121	*<LOD*	*<LOD*	0.076 ± 0.011	0.470 ± 0.048	0.439 ± 0.116	0.227 ± 0.076
		Virulent	0.858 ± 0.194	*<LOD*	*<LOD*	*<LOD*	**0.371 ± 0.083** *	*<LOD*	**0.474 ± 0.133** ***
	Root	*Control*	0.740 ± 0.198	*<LOD*	*<LOD*	0.063 ± 0.014	0.092 ± 0.011	0.340 ± 0.081	0.285 ± 0.082
		Avirulent	0.635 ± 0.047	*<LOD*	*<LOD*	**0.141 ± 0.020** ***	**0.123 ± 0.008** *	**0.198 ± 0.051** *	**0.173 ± 0.034** *
		Virulent	0.612 ± 0.085	*<LOD*	*<LOD*	0.078 ± 0.017	0.197 ± 0.018 ***	*<LOD*	0.346 ± 0.072

Timepoint	Tissue	Status	Total cZ-types		cZ		cZOG		cZR		cZROG		cZRMP		cZ9G	
4 hours post-inoculation	Cotyledon	*Control*	1.05 ± 0.09		0.16 ± 0.03		*< LOD*		0.89 ± 0.09		*< LOD*		*< LOD*		*< LOD*	
		Avirulent	**0.40 ± 0.06**	***	0.17 ± 0.03		*< LOD*		**0.24 ± 0.04**	***	*< LOD*		*< LOD*		*< LOD*	
		Virulent	**0.52 ± 0.13**	***	**0.24 ± 0.06**	*	*< LOD*		**0.28 ± 0.07**	***	*< LOD*		*< LOD*		*< LOD*	
2 days post-inoculation	Cotyledon	*Control*	9.16 ± 2.06		0.15 ± 0.03		1.26 ± 0.14		0.65 ± 0.03		0.74 ± 0.22		10.40 ± 1.23		*< LOD*	
		Avirulent	7.62 ± 0.95		**0.84 ± 0.28**	***	1.52 ± 0.33		0.74 ± 0.23		**0.32 ± 0.05**	***	**7.42 ± 2.24**	*	*< LOD*	
		Virulent	8.30 ± 2.66		**0.43 ± 0.12**	***	1.25 ± 0.18		0.64 ± 0.09		0.52 ± 0.10		**7.09 ± 1.86**	*	*< LOD*	
5 days post-inoculation	Cotyledon	*Control*	19.85 ± 2.84		0.19 ± 0.02		0.98 ± 0.12		0.77 ± 0.09		0.66 ± 0.07		17.25 ± 2.69		*< LOD*	
		Avirulent	25.29 ± 4.19		**1.32 ± 0.25**	***	0.91 ± 0.16		**1.39 ± 0.07**	**	0.76 ± 0.18		20.90 ± 4.24		*< LOD*	
		Virulent	17.06 ± 3.20		**0.58 ± 0.14**	***	**1.35 ± 0.21**	*	0.97 ± 0.22		0.74 ± 0.12		13.42 ± 2.71		*< LOD*	
	Shoot	*Control*	150.22 ± 7.05		2.31 ± 0.30		2.54 ± 0.40		12.41 ± 2.26		12.30 ± 1.48		120.66 ± 3.87		*< LOD*	
		Avirulent	190.31 ± 42.20		**4.05 ± 0.24**	**	2.27 ± 0.32		**5.37 ± 0.35**	***	**5.97 ± 0.84**	***	**172.65 ± 41.12**	*	*< LOD*	
		Virulent	**73.81 ± 4.47**	***	**1.63 ± 0.21**	*	**3.67 ± 0.24**	**	**2.95 ± 0.41**	***	**5.82 ± 0.27**	***	**59.75 ± 4.54**	***	*< LOD*	
	Root	*Control*	71.79 ± 11.97		2.10 ± 0.15		2.83 ± 0.16		5.27 ± 0.09		13.04 ± 1.41		48.25 ± 10.84		0.34 ± 0.02	
		Avirulent	**108.39 ± 17.20**	*	**5.94 ± 0.67**	***	**4.11 ± 0.64**	*	**7.87 ± 0.74**	*	10.22 ± 2.07		**79.42 ± 13.73**	*	**0.83 ± 0.05**	***
		Virulent	66.46 ± 3.22		**3.23 ± 0.30**	***	**3.58 ± 0.15**	**	5.02 ± 0.25		13.23 ± 0.93		40.79 ± 3.14		**0.61 ± 0.10**	**
11 days post-inoculation	Cotyledon	*Control*	48.64 ± 10.00		1.01 ± 0.18		1.76 ± 0.41		1.57 ± 0.22		1.29 ± 0.37		43.00 ± 9.09		*< LOD*	
		Avirulent	**70.15 ± 4.58**	***	**4.41 ± 0.85**	***	**8.63 ± 1.99**	***	**2.09 ± 0.19**	*	**3.50 ± 0.95**	***	51.52 ± 2.33		*< LOD*	
		Virulent	**11.55 ± 0.94**	***	0.86 ± 0.17		**1.19 ± 0.13**	*	**0.39 ± 0.02**	***	1.21 ± 0.11		**7.91 ± 1.10**	***	*< LOD*	
	Shoot	*Control*	40.01 ± 7.48		0.69 ± 0.08		2.52 ± 0.40		1.97 ± 0.28		3.84 ± 0.47		30.98 ± 6.60		*< LOD*	
		Avirulent	**62.13 ± 4.05**	***	**1.40 ± 0.14**	***	**5.02 ± 0.56**	***	**2.97 ± 0.28**	*	4.67 ± 0.56		**48.07 ± 3.51**	***	*< LOD*	
		Virulent	**25.85 ± 6.14**	*	**1.61 ± 0.51**	***	3.36 ± 0.08		**1.23 ± 0.32**	*	3.82 ± 0.20		**21.83 ± 4.31**	*	*< LOD*	
	Root	*Control*	73.93 ± 9.87		1.25 ± 0.20		3.54 ± 0.57		3.59 ± 0.45		9.48 ± 0.81		55.83 ± 9.00		0.24 ± 0.01	
		Avirulent	67.64 ± 3.19		**3.93 ± 0.91**	***	**5.70 ± 0.53**	**	2.67 ± 0.44		11.17 ± 2.21		44.05 ± 4.15		**0.15 ± 0.03**	*
		Virulent	**37.44 ± 0.55**	**	1.85 ± 0.56		**2.11 ± 0.32**	*	**1.44 ± 0.05**	**	7.76 ± 0.50		**24.24 ± 0.23**	**	**0.04 ± 0.00**	***
15 days post-inoculation	Cotyledon	*Control*	36.37 ± 1.53		0.62 ± 0.10		9.63 ± 0.32		2.66 ± 0.48		8.39 ± 0.69		15.07 ± 1.84		*< LOD*	
		Avirulent	41.95 ± 5.85		**1.04 ± 0.06**	*	**2.96 ± 0.36**	***	2.52 ± 0.20		**2.42 ± 0.42**	***	**33.01 ± 5.01**	***	*< LOD*	
		Virulent	34.04 ± 6.35		0.70 ± 0.10		**1.63 ± 0.36**	***	2.97 ± 0.48		**2.81 ± 0.70**	***	**25.93 ± 5.06**	**	*< LOD*	
	Shoot	*Control*	28.52 ± 1.18		0.76 ± 0.13		2.93 ± 0.17		3.02 ± 0.75		4.05 ± 0.80		17.76 ± 0.78		*< LOD*	
		Avirulent	**40.99 ± 4.96**	**	**2.50 ± 0.45**	***	3.16 ± 0.09		**2.23 ± 0.13**	*	3.32 ± 0.29		**29.79 ± 5.02**	**	*< LOD*	
		Virulent	27.94 ± 2.93		**1.08 ± 0.19**	*	2.54 ± 0.31		2.92 ± 0.82		4.07 ± 0.73		17.33 ± 1.75		*< LOD*	
	Root	*Control*	46.24 ± 4.70		1.20 ± 0.05		2.24 ± 0.20		5.50 ± 0.93		12.50 ± 2.93		24.46 ± 3.03		0.34 ± 0.08	
		Avirulent	**81.49 ± 7.11**	***	**6.30 ± 0.31**	***	**4.02 ± 0.11**	***	5.57 ± 0.40		14.44 ± 2.17		**50.85 ± 5.76**	***	0.32 ± 0.07	
		Virulent	**60.85 ± 5.49**	**	**7.20 ± 0.42**	***	**3.19 ± 0.41**	***	4.46 ± 0.28		10.44 ± 1.84		**34.91 ± 3.36**	*	**0.65 ± 0.13**	**
25 days post-inoculation	Cotyledon	*Control*	17.91 ± 1.39		0.68 ± 0.14		4.45 ± 0.78		1.19 ± 0.31		6.22 ± 1.56		6.34 ± 1.11		*< LOD*	
		Avirulent	**26.60 ± 2.63**	***	**3.40 ± 0.41**	***	4.06 ± 0.54		**3.77 ± 0.88**	***	5.10 ± 0.73		**10.28 ± 1.77**	**	*< LOD*	
		Virulent	14.99 ± 2.00		**1.44 ± 0.37**	**	**2.23 ± 0.29**	***	1.55 ± 0.31		4.57 ± 1.00		5.20 ± 0.78		*< LOD*	
	Shoot	*Control*	17.34 ± 0.19		0.91 ± 0.10		3.15 ± 0.33		1.47 ± 0.30		2.58 ± 0.30		9.23 ± 0.50		*< LOD*	
		Avirulent	**20.51 ± 0.97**	**	1.04 ± 0.06		3.27 ± 0.18		1.30 ± 0.29		2.92 ± 0.18		**11.98 ± 0.81**	***	*< LOD*	
		Virulent	**27.21 ± 5.31**	***	0.85 ± 0.14		2.65 ± 0.37		**3.81 ± 1.24**	**	**5.22 ± 1.61**	**	**14.68 ± 3.20**	**	*< LOD*	
	Root	*Control*	63.39 ± 1.66		5.46 ± 0.90		3.77 ± 0.45		4.51 ± 0.33		28.85 ± 3.92		19.99 ± 3.08		0.81 ± 0.10	
		Avirulent	63.89 ± 4.17		6.34 ± 1.12		3.93 ± 0.68		5.26 ± 0.22		27.80 ± 3.82		19.83 ± 1.35		0.73 ± 0.10	
		Virulent	**48.14 ± 1.21**	***	**1.24 ± 0.05**	***	**2.59 ± 0.08**	***	**3.08 ± 0.36**	***	21.54 ± 1.24	**	19.31 ± 1.22		**0.39 ± 0.10**	***
