# Supplementary material for: The Cytokinin Complex Associated With Rhodococcus fascians: Which Compounds Are Critical for Virulence?
Source: Front Plant Sci. 2019 May 22;10:674. doi: 10.3389/fpls.2019.00674 (PMC6539147; doi:10.3389/fpls.2019.00674)
Supplement: Supplementary file 2 [file Table_2.pdf]

Table S2: *Rhodococcus fascians* genes detected in various *Rhodococcus fascians* isolates

Sl.No	Strain	*mt1*	*mt2*	*dprA*	*16S*	*fas4*	Virulence classification[a]
1	591	+	+	+	+	+	V
2	592	+	+	+	+	+	V
3	594 vir 1	+	+	+	+	+	V
4	595	+	+	+	+	+	V
5	596	+	+	+	+	+	V
6	598	+	+	+	+	+	V
7	599 vir 2	+	+	+	+	+	V
8	600	+	+	+	+	+	V
9	601	+	+	+	+	+	V
10	602	+	+	+	+	+	V
11	603	+	+	+	+	+	V
12	604	+	+	+	+	+	V
13	605	+	+	+	+	+	V
14	610	+	+	+	+	+	V
15	665	+	+	+	+	+	V
16	666 vir 3	+	+	-	+	+	V
17	606 vir 4	+	+	-	+	+	V
18	670	+	+	+	+	+	V
19	672	-	-	-	+	-	a
20	676	-	-	-	+	-	a
21	677	-	-	-	+	-	a
22	589	-	-	-	+	-	a
23	664	-	-	-	+	-	a
24	668	-	-	-	+	-	a
25	671	-	+	+	+	-	a
26	674	-	-	+	+	-	a
27	593 avir	-	-	-	+	-	a
28	590	-	+	-	+	-	a
29	667	-	+	-	+	-	a
30	669	-	-	-	+	-	a
31	597	-	-	-	+	-	a
32	609	-	-	-	+	-	a
33	673	-	-	-	+	-	a
34	675	-	-	-	+	-	a
35	607	-	-	-	+	-	a
36	608	-	-	-	+	-	a
37	*Rhodococcus sp.* Vir 5	+	+	+	+	+	V
38	*Williamsia sp.* Willi	+	+	+	+	+	V

[a]See Stange et al. (1996)
