# Supplementary material for: The Cytokinin Complex Associated With Rhodococcus fascians: Which Compounds Are Critical for Virulence?
Source: Front Plant Sci. 2019 May 22;10:674. doi: 10.3389/fpls.2019.00674 (PMC6539147; doi:10.3389/fpls.2019.00674)
Supplement: Supplementary file 4 [file Table_4.pdf]

Table S4. ***Rhodococcus fascians*** **strains used in this study and their details**

**Strain**	**Origin**	**Remarks**
589	PDDCC 260	From ICPB[b] CF1 Hilderbrand USA.
590	PDDCC 2603	From ICPB CFI5 Davey Canada.
591	PDDCC 2604	From ICPB CFI9 Tilford USA. ATTCC[d] 12975.
592	PDDCC 2605	From ICPB CF21 Tilford USA.
593	PDDCC 2606	From ICPB CFI01 Domdroff USA.
594	PDDCC 5340	Origin sweet pea shoot, Taylor UK.
595	PDDCC 5833	Burkholder CF17 Tilford ATCC 12974 NCPB 3067-1
596	PDDCC 7108	Origin *Verbascum nigrum* Miller 1978, causes leafy gall on bulblet.
597	PDDCC 7109	Origin *Gladiolus* Miller 1978, causes leafy gall on bulblet.
598	PDDCC 7112	Origin *Brodiea laxa* Miller 1978, causes leafy gall on bulblet.
599	PDDCC 7113	Origin *Begonia* Van Hoof 1977, causes organoid tumours on roots.
600	PDDCC 7114	Origin Dahlia stem base Van Hoof 1978.
601	PDDCC 6788	Origin *Carica pubescens* Watson 1980, Bud proliferation.
602	PDDCC 6789	Origin *Carica pubescens* Watson 1980, Bud proliferation.
603	PDDCC 6790	Origin *Carica pubescens* Watson 1980, Bud proliferation.
604	PDDCC 6791	Origin *Carica pubescens* Watson 1980, Bud proliferation.
605	PDDCC 6792	Origin *Carica babaco* Watson 1980, Subterranean proliferation of buds on cuttings.

606	PDDCC 6793	Origin *Carica babaco* Watson 1980, Subterranean proliferation of buds on cuttings.
607	PDDCC 7340	Origin *Phytolacca octandra* Watson 1980, Bud proliferation.
608	PDDCC 7364	Origin *Dahlia* Watson 1980, Bud proliferation on tubers.
609	6D-21	Kado 85 Dr. Anne Vidaver U of Nebraska Lincoln.
610	79-1	Kado 85 Dr. Anne Vidaver U of Nebraska Lincoln.
664	NCPPB 156	Origin *Chrysanthemum morifolium* Jacobs 1945 UK.
665	NCPPB[c] 188	Origin *Chrysanthemum morifolium* Dowson 1946 UK. Reisolate of NCPPB 156
666	NCPPB 469	Origin *Fragaria chiloensis* Lelliott 1975 UK.
667	NCPPB764	Origin *Fragaria chiloensis* (symptomless) .Crosse 1951 UK.
668	NCPPB765	Origin *Fragaria chiloensis* (symptomless) .Crosse 1951 UK.
669	NCPPB 766	Origin *Fragaria chiloensis* (symptomless) .Crosse 1951 UK.
670	NCPPB 1488	Origin sweet pea Lelliot 1963 UK. ICPB CF115S.
671	NCPPB 1675	Origin *Chrysanthemum morifolium* Jones 1964 UK.
672	NCPPB 1733	Origin *Beloperone guttata* Oxtoby 1965 UK.
673	NCPPB 2210	Origin *Tulipa gesneriana* Catton 1969 UK.
674	NCPPB 2554	Origin *Phlox sp*. Baker 1973 UK.
675	NCPPB 2555	Origin *Mesembryanthum sp*. Baker 1973 UK.
676	NCPPB 2556	Origin *Petunia sp*. Baker 1973 UK.
677	NCPPB 2557	Origin *Verbena sp*. Baker 1973 UK.

a PDDCC Plant Disease Division Culture Collection, NZ.
b ICPB International Collection of Phytopathogenic Bacteria, USA.
c NCPPB National Collection of Plant Pathogenic Bacteria, UK.
\- no culture collection number.
Source: Eason, 1993
