# Supplementary material for: The Cytokinin Complex Associated With Rhodococcus fascians: Which Compounds Are Critical for Virulence?
Source: Front Plant Sci. 2019 May 22;10:674. doi: 10.3389/fpls.2019.00674 (PMC6539147; doi:10.3389/fpls.2019.00674)
Supplement: Supplementary file 5 [file Table_5.pdf]

Table S5. ***Rhodococcus fascians*** **primers and reference genes used in PCR and RT-qPCR**

**Gene Name**	**Sequences of Primers**	**Accession No.**
Rfmt1F2<br>*Rfmt1R2*	TCCTTTGCCGCCCAACTCTC<br>GGCGTACATCGTCGTCTCGT	JN093097
Rfmt2F1<br>*Rfmt2R1*	CCGGATGGAGTCGCTGGAAATC<br>GCCTGAACCATGTTTGCGAAGTG	JN093097
Rffas4F4<br>*Rffas4R5*	GAGTTCGCCTTCTCCCATTTC<br>CGACAGCACCGCATCTAAAC	Z29635
RfdprAF1<br>*RfdprAR1*	GGATGGCTGGAGGGATCGACTC<br>GCAACACCGCTCCCTCGATC	AF001836
Rf16SF2<br>*Rf16SR2*	TTGTCTTATGTTGCCAGCACGTAATG<br>CGATTACTAGCGACTCCGACTTCAC	Y11196
18SF2<br>*18SR2*	GCTGAAACTTAAAGGAATTGACGGAAG<br>TTGAAGACCAACAATTGCAATGATCTATC	
PsGAPDHF<br>*PsGADHR*	GGTATGTCATTCCGTGTCCCA<br>CCCTCAGACTCTTCCTTGATAGC	
