# Supplementary material for: The Cytokinin Complex Associated With Rhodococcus fascians: Which Compounds Are Critical for Virulence?
Source: Front Plant Sci. 2019 May 22;10:674. doi: 10.3389/fpls.2019.00674 (PMC6539147; doi:10.3389/fpls.2019.00674)
Supplement: Supplementary file 6 [file Image_1.pdf]

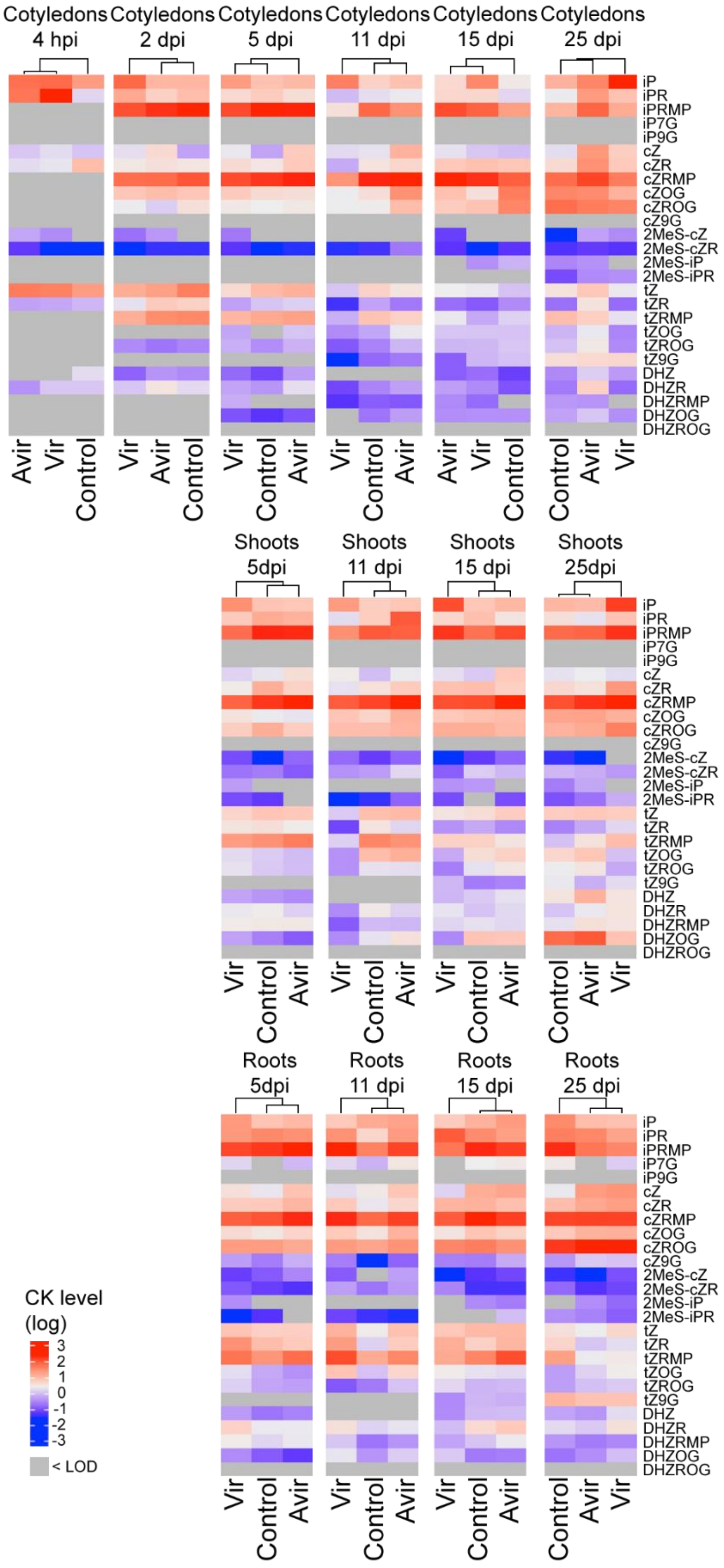

Supplementary Figure 1. Abundance of cytokinins in cotyledons, shoots and roots of peas inoculated with different *R. fascians* strains. Heatmaps were constructed using log-transformed

data of cytokinin content in cotyledons at 4 hours post-inoculation (hpi) and 2 days post inoculation (dpi), and in cotyledons, roots and shoots at 5 dpi, 11 dpi, 15 dpi, and 25 dpi with the avirulent strain 589 (Avir), the virulent strain 602 (Vir), and mock-inoculated control. Clustering of treatments per tissue and time point was performed using Pearson correlation coefficients for the distance matrix and average linkage as the clustering method, with bootstrapping of 1000 iterations. Values below the limit of detection (< LOD) or not determined (n.d.) are depicted in gray. Data for iP-types and Z-types is published in Dhandapani et al. (2018, 2019).
