# Supplementary material for: The Cytokinin Complex Associated With Rhodococcus fascians: Which Compounds Are Critical for Virulence?
Source: Front Plant Sci. 2019 May 22;10:674. doi: 10.3389/fpls.2019.00674 (PMC6539147; doi:10.3389/fpls.2019.00674)

Supplementary Figure 2: Photos of pea seed cv Onward inoculated with *Williamsia* Leaf354 (left) and Rhodococcus Leaf225 (right)

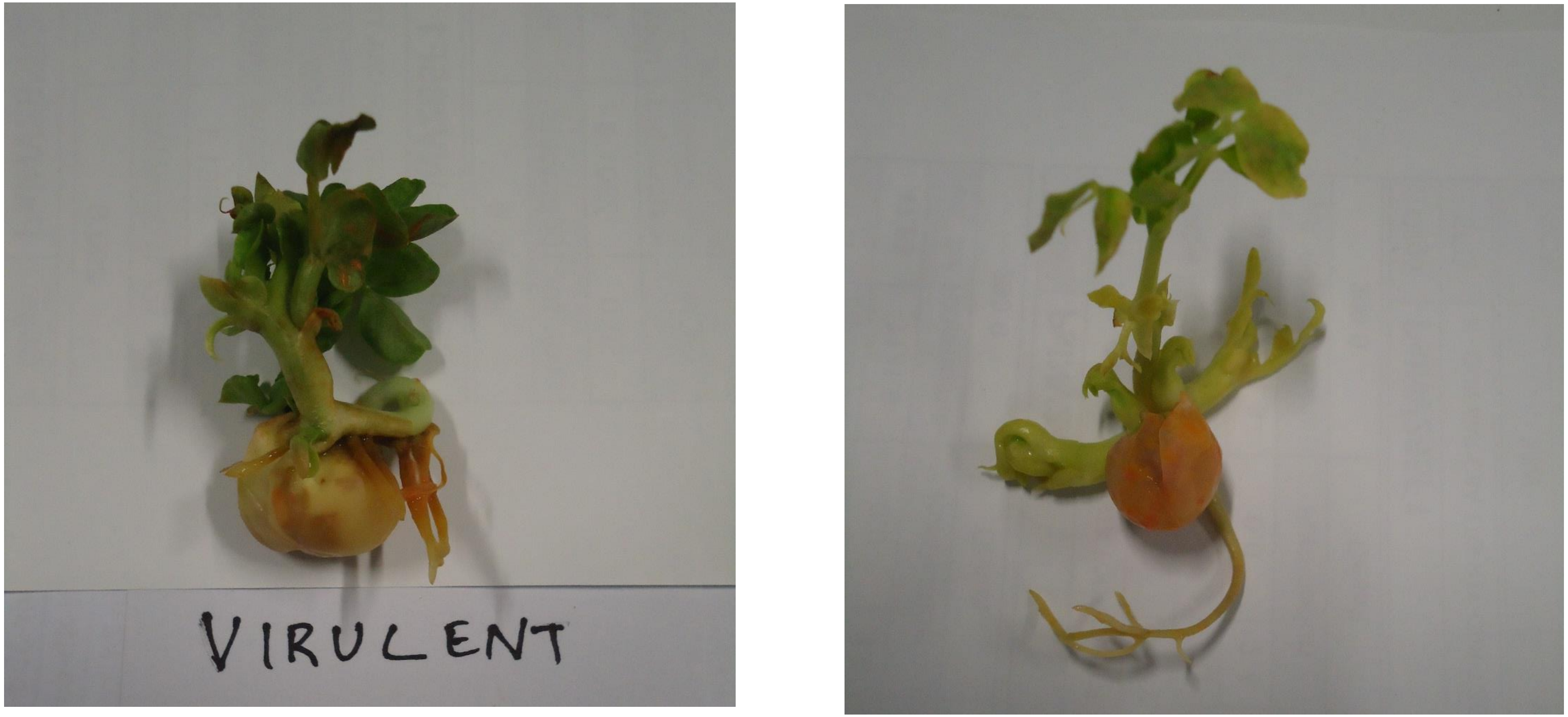

Supplement: Supplementary file 7 [file Image_2.pdf]
