# Supplementary material for: The Cytokinin Complex Associated With Rhodococcus fascians: Which Compounds Are Critical for Virulence?
Source: Front Plant Sci. 2019 May 22;10:674. doi: 10.3389/fpls.2019.00674 (PMC6539147; doi:10.3389/fpls.2019.00674)
Supplement: Supplementary file 8 [file Image_3.pdf]

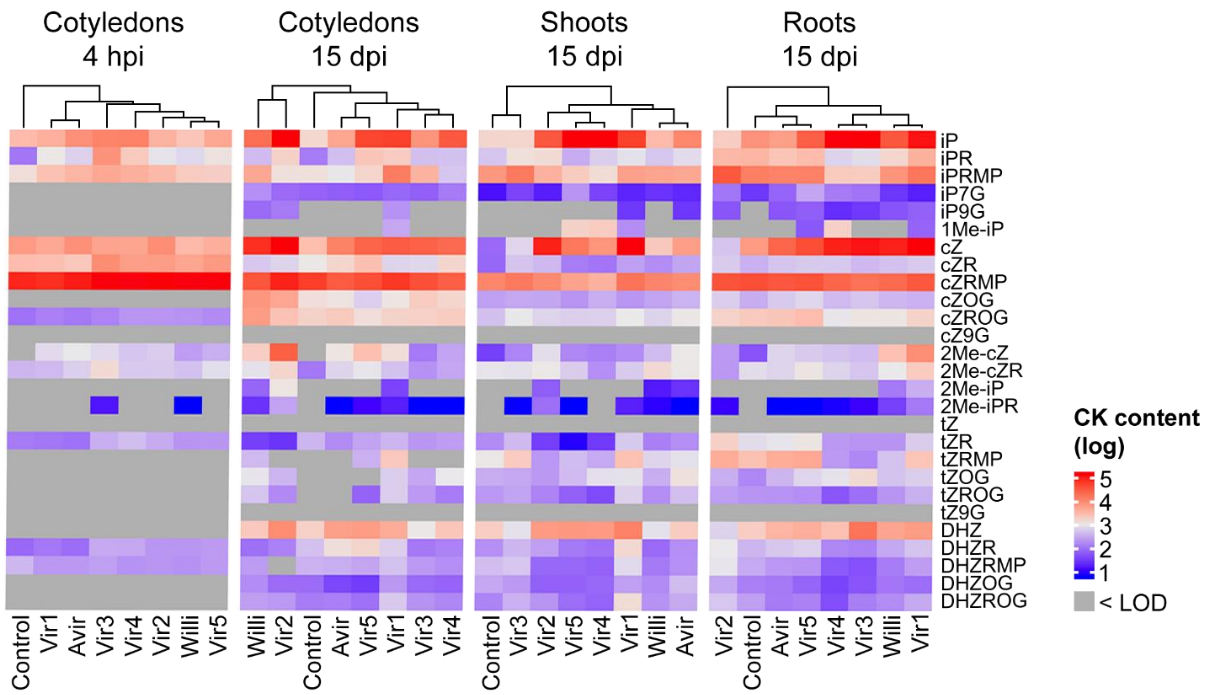

Supplementary Figure 3. Abundance of cytokinins in cotyledons, shoots and roots of peas inoculated with different *R. fascians* and *Williamsia* sp strains. Heatmaps were constructed using log-transformed data of cytokinin content in in cotyledons at 4 hours post-inoculation (hpi), and in cotyledons, shoots and roots at 15 days post inoculation (dpi) with an avirulent strain Avir, six different virulent strains, and mock-inoculated control. Strains used are as described in Stange et al. (1996): Avir: avirulent strain 593; Vir1: virulent strain 594 (similar 210 kb linear plasmid to virulent strain 602); Vir2: 599 (same RFLP pattern to 602 but linear plasmid not detected, no DprA detected); Vir3: 666 (circular plasmid); Vir4: 606 (130 kb linear plasmid). Vir5: *R. fascians* sp. Leaf225 isolated from non-symptomatic arabidopsis leaves; and Willi: *Williamsia* sp. Leaf354, also isolated from non-symptomatic arabidopsis leaves. Clustering of treatments per tissue and time point was performed using Pearson correlation coefficients for the distance matrix and average linkage as the clustering method, with bootstrapping of 1000 iterations. Values below the limit of detection (< LOD) are depicted in gray.
